# Ierne Propounds Causes and a Remedy

**Published:** 1919-03-08

**Authors:** 


### TRADE UNIONS AND NURSES.

### Ierne Propounds Causes and a Remedy.

Something quite remarkable has happened in Ireland. As readers of The Hospital are aware, there are many factions even in nursing circles there, as well as never ceasing activity amongst the "leaders," but so far as the working nurse is concerned Ireland has been, and is, a veritable Sleepy Hollow. Nobody has ever made even a show of effort to better her lot. When public meetings are held, which is seldom, she is not talked with but at. She bears the same relation to her "leaders" that the fish in the sea bear to a battleship that floats on the surface, and if any of the "leaders" decided to demobilise their warring camps and to disappear, in Mr. Gilbert's language, "they never would be missed." I regret to have to say that to this there is no exception. During the present generation none of the "leaders" can lay claim to any achievement that has brought relief or pleasure or gain or other tangible benefit to the hard-worked and ill-paid woman whose cause they profess to champion.

Enter Miss Louie Bennett.

Into this desert, silently and suddenly, like an apparition, enters Miss Louie Bennett, Organising Secretary of the Irish Women Workers' Union, who with the consent and sympathy of the Lord Mayor of Dublin, summoned a public meeting with the object of forming an Irish Nurses' Trades Union. Miss Louie Bennett is a woman of strong personality, a prominent suffragist, an organiser of skill and will, and her services were solicited by some members of the profession who have abandoned all hope of succour from within the fold. On Friday of last week this meeting was held, and it would be untrue to say that it was not a success. It was appallingly successful. "I came with an open mind," said a well-known and representative nurse to me when the meeting ended, "and I go away a convinced Trades Unionist. I have been listening to our own people all my life, and they have never done or tried to do a single thing to better our condition."

Miss Bennett explained her purpose without verbiage and with perfect clearness. The I.W.W.U., she said, is non-political and non-sectarian. It has one sole object, namely, to improve the working conditions of women in Ireland. A Nurses' Union, if formed, will be a completely independent branch except in the matter of finance. Nurses will be invited to form their own Committee and control their own business in every way. As regards a strike Miss Bennett emphasised that such is not only wholly unnecessary, but altogether unthinkable for nurses. The I.W.W.U. had only once during the past two years advised a strike, and yet the benefits that it had brought to its members amount to something like £1,000 a week. Dr. Hennessy followed, and said that he had seen nurses on duty for fourteen hours; he had seen them do charwomen's work, and if any nurses were so snobbish as to think that they were lowering their professional dignity by joining a Trades Union they could go their way and let their grievances remain unredressed. Several other speakers addressed the meeting, including representatives of clerks and journalists showing how by joining a Trades Union their wages and privileges had been substantially increased. A letter expressing regret for non attendance, but full sympathy with a Nurses' Trades Union, was read from the Hon. Albinia Brodrick. Towards the close of the meeting feeling was beginning to run high, and such words as "liar" were audibly whispered. A considerable number of nurses enrolled, and Miss Bennett closed the meeting with the exhortation not to be in a hurry—to think the position well over—and announced that another public meeting will be held in the early future.

The Nursing Position in Ireland.

This, in brief outline, is the situation. I have been accused of hustling and alarmist tendencies, but perhaps these occurrences will convince the most sceptical that my warnings were timely. The question arises, What is to be done? This question has, indeed, already arisen, and secret and anxious meetings are being held in Dublin. It has suddenly been realised that all the shibboleths and fiery academic discussions about visionary legislation and limited companies and by interference and employers' societies are as the crackling of thorns under a pot. They do not matter one tiny atom. The nurses have gone outside in search of a champion, and Miss Bennett has answered the call.

Can the Situation be saved by the College?

If the situation is to be saved by the College—in Great Britain, I mean—immediate action must be taken. What is urgently required is that intangible and wonderful thing called Imagination. Pardon my saying so, but it is singularly lacking in the kingdom of the nurse. For example, on the eve of the Dublin meeting, the Dublin Branch of the College of Nursing passed a series of resolutions in favour of better employment conditions for Irish nurses. One of these was that there should be one day off in fourteen. May I ask where the suggestion finds its origin? To my mind, it shows an extraordinary lack of imagination, a sort of conviction that the British people demand a sacrifice from nurses that no other section of the community is called upon to make. Again, on the eve of this meeting Miss Vera Matheson issues a pamphlet setting out very ably the wrongs from which Irish nurses suffer. But all through there is a note of reproach that the public have been so illiberal. "We don't want to subscribe money to voluntary hospitals in order that they may pay their nursing staff adequately. It doesn't appeal to us, and so we don't do it." Herein is a fallacy. The British public have never yet been asked to do what Miss Matheson says they don't want to do. I have read scores of hospital reports, most of them containing long lists of names of magnanimous subscribers. In these reports I have never yet seen a note expressing that the nurse is ill paid and hard worked. *Vide* Lord Knutsford's Charter of Liberty and his comments on the growing tendency to growl. Here is the Dublin Branch of the College of Nursing asking that the nurse should have one day off duty in fourteen. This lack of confidence in the public is peculiar to nursing.

The Position of Nurses in the United States.

Precisely the same condition of affairs exists in America. A writer in a recent issue of the *American Journal of Nursing* points out that nursing reforms have "usually come from people outside the established order, because the people who were doing the work could not see the abuses which had become intolerable, or had not the strength or courage to rise and sweep them out of existence."

The Lack of Imagination to Check Trades Unionism.

What is lacking is imagination. The bonds of tradition must be broken, and the case of the nurse must be put before the public, not in a niggardly and half-hearted way, but in full confidence that no reasonable request will be refused. Pay that will attract the best women, a reasonable day's work, one whole day and one half-day off duty in seven. Make this a demand; make it with boldness and confidence; make it at once, and, if necessary, appeal to the supreme centre of British public opinion, the House of Commons.

That is the only way to checkmate Trades Unionism.

IERNE.

